# Praxis, Power, and Processes: Youth Participation in Health Policy – A Response to Recent Commentaries

**DOI:** 10.34172/ijhpm.2024.8567

**Published:** 2024-05-21

**Authors:** Tanya Jacobs, Asha George

**Affiliations:** School of Public Health, Faculty of Community and Health, University of the Western Cape, Cape Town, South Africa.

 We are appreciative of the opportunity to further engage in critical discussions and to advance the knowledge on youth participation in health policy processes, by providing a response to commentaries on our paper, “Between rhetoric and reality: learnings from youth participation in the Adolescent and Youth Health Policy (AYHP) in South Africa.”^1^

 The commentaries by Njelesani & Hunleth^2^, Prati & Albaseni,^3^ and O’Connell & Botchway^4^ all add valuable reflections and contributions in addressing the gap between rhetoric and reality of youth participation, the key theme explored in our paper. We highlight the main threads across our paper and the three commentaries in the sections below focusing on interrelated elements of praxis, power and policy processes.

## Praxis – Applying Theoretical Approaches, Frameworks and Disciplinary Lenses

 A key thread across the commentaries is the need to unpack and analyse key concepts and buzzwords such as “participation,” “youth,” and “meaningful youth participation” that are sometimes used in uncritical waysin both global and national discourses related to youth participation across key challenges such as health, climate change and working towards gender equality. Further, policy discourses about youth can be somewhat contradictory, constructing young people simultaneously as both ‘a risk’ to social cohesion and democracy and “a solution” to “wicked problems.”

 Moving beyond rhetoric will require that these terms and buzzwords be critically examined as part of our praxis, both in terms of definitions and strategies to integrate them in policy processes. We agree with the key insights and reflections highlighted by the commentaries, for example Njelesani and Hunleth^2^ provide insights on how our conceptual framework can be used to advance youth participation to inform equitable health policies, including the inclusion of youth with intersecting identities, such as those living with disabilities. In addition, they make contributions by juxtaposing questions from their Reflective Guide to the list of prospective questions to guide youth participation in policy processes from our paper, to delve even deeper into issues of meaningful inclusion in research and policy development within the contexts in which young people live.

 In terms of praxis ie, applying theories and frameworks from a range of disciplines, we advocate for building on existing scholarship to advance the field, as we were inspired by the opportunity to expand and build on the conceptual framework of Cahil and Dadvand,^5^ synergized from fields of feminist, post-structural and critical theory, as well as youth studies and citizenship research into youth participation. As scholars grounded in Health Policy and Systems Research our contribution foregrounded the interactions, frameworks, and ideas about policy processes.^6-10^ We explored the dynamic relationships which exist between policy contexts, actors, content, and processes as part of our analysis of AYHPs in South Africa, with the disconnect between rhetoric and reality of youth participation emerging as a key finding, the catalyst for the paper.

 Also, as noted by Prati and Albanseni an understanding of literatures on “transformative partnerships”^11^ and “co-production” will be helpful in studying ways of youth participation that might not be recognized by conventional understandings of participation and in theories and frameworks from youth studies. A cross-cutting message across our work is that to further advance the scholarship and praxis related to youth participation in health policy we need to engage and synergize a range of disciplines focusing on policy processes that facilitate meaningful participation, as well as actor and power analyses.^12-14^

## Power

 A second theme illustrated in our paper as well as the commentaries is that of power, and that the work of youth participation is not merely technical and a tick-box exercise, but deeply political, because it has to do with power relations at many different levels. Our paper as well as the commentaries, highlight the importance of understanding power relations embedded in social and political contexts and how these shape participation processes. Firstly, we appreciate the points underscored by Njelesane and Hunleth^2^ that power relations in the nature and quality of relationships between youth and adults are critical to the success of participation processes and outcomes. This requires critical reflections and transformation of relationships between youth and adults and the alignment of agendas should be all our business.

 Secondly, youth are not homogenous and the social and structural systems of power shape both their health as well as their ability to engage with adolescent and youth policy processes, not only in South Africa but in other contexts and this remains a critical area for ongoing research. Another insightful learning is from disability studies and documented by Peta^15^ and Ngunyen et al,^16^ who describe how girls living with disabilities can participate in policy processes.

 In response to the question raised by Njelesani and Hunleth as to how we included diverse youth, we did not intend to interview perspectives of representative and diverse youth and structures in the general population, but wanted to have views of diverse policy actors directly and indirectly involved in the AHYP policy development process. Intersectional systems of inequality and discrimination based on gender, race, class, ability, sexual orientation and gender identity etcare very important to foreground when engaging in debates in the context of multiple actors, power relations and inequalities.^17-19^ In addition, we agree with the learnings from youth participation in climate change as relevant to youth participation in health policy, as asserted by O’Connell and Botchway.^4^ They note that relationship building is essential, it creates intergenerational learning, and that tokenism challenges the participatory process and reinforces power relations.

 A key message from our findings, as well as the commentaries, is the importance of moving to more systematic processes of routinely including the voices and agency of young people, in their full diversity, in all policies and programmes, which remains both an ambitious goal and a vexing challenge to implement in reality. This will include understanding intersectionality and applying the approaches to integrate perspectives of diverse young people as an essential component of youth participation in policy process, otherwise we just reproduce power relations.

## Policy Processes – Participation as a Right

 A third key theme is that participation in policy processes is a right and it should be a priority to involve youth voices as they can they make significant contributions and provide leadership in both programme and policy processes and meaningful engagement leads to healthier, more just, and equal societies.^20-22^ Prati and Albanesi^3^ foreground critical questions on why youth participation is a right and a requirement for the sake of youth themselves, as well as policies and programmes by asking for example how we define and who is included and excluded. We agree with their call for further research on youth participation through the theoretical lens of transformative participation and unpacking adult-youth relationships, particularly youths’ voice and perceptions of their experience.^23-25^

 Policy processes for youth participation underscores the necessity to strengthen capacities, necessary platforms and the training, ongoing mentorship needed, as also highlighted by others.^26,27^ As policy-makers, researchers and young people, we need to prioritize the competency gaps and determinants of youth participation to ensure sustained, deep and meaningful ways, beyond the rhetoric of a few token young people and “older” experts in policy processes. In addition to the enabling contexts and organisational architecture, our findings a well as the three commentaries reiterate the need for shifts in mindsets, paradigms, developing innovative partnerships and capacity strengthening for ethical youth engagement at national and global levels.^28,29^

## Conclusion

 Policy-makers need to meaningfully engage youth in their diversity and in representative and accountable ways, in all stages and spaces of the policy-making process, as part of building youth citizenship and leadership. Looking ahead, an essential element is a mobilized, capacitated, diverse youth citizenry as important actors to ensure youth participation, and the use of available tools and resources and guidance in a reflexive manner. To bridge the gap between rhetoric and reality, we amplify the call for reimagining of new paradigms, policy processes and transformation of systems of power.

## Ethical issues

 This correspondence is based on an article which was part of a larger PhD research study titled: People, power and processes: a gender analysis of adolescent health policy in South Africa which has received ethical approval by the Biomedical Science Research Ethics Committee of the University of the Western Cape. Reference number: BM18/9/9.

## Competing interests

 Authors declare that they have no competing interests.
